# Comparison of CT images with average intensity projection, free breathing, and mid‐ventilation for dose calculation in lung cancer

**DOI:** 10.1002/acm2.12037

**Published:** 2017-01-24

**Authors:** Chirasak Khamfongkhruea, Sangutid Thongsawad, Chirapha Tannanonta, Sasikarn Chamchod

**Affiliations:** ^1^ Radiation Oncology Department Chulabhorn Hospital Chulabhorn Royal Academy of Science Bangkok Thailand

**Keywords:** 4DCT, average intensity projection, dose calculation, lung cancer, mid‐ventilation

## Abstract

The purpose of this study was to compare three computed tomography (CT) images under different conditions—average intensity projection (AIP), free breathing (FB), mid‐ventilation (MidV)—used for radiotherapy contouring and planning in lung cancer patients. Two image sets derived from four‐dimensional CT (4DCT) acquisition (AIP and MidV) and three‐dimensional CT with FB were generated and used to plan for 29 lung cancer patients. Organs at risk (OARs) were delineated for each image. AIP images were calculated with 3D conformal radiotherapy (3DCRT) and intensity‐modulated radiation therapy (IMRT). Planning with the same target coverage was applied to the FB and MidV image sets. Plans with small and large tumors were compared regarding OAR volumes, geometrical center differences in OARs, and dosimetric indices. A gamma index analysis was also performed to compare dose distributions. There were no significant differences (*P* > 0.05) in OAR volumes, the geometrical center differences, maximum and mean doses of the OARs between both tumor sizes. For 3DCRT, the gamma analysis results indicated an acceptable dose distribution agreement of 95% with 2%/2 mm criteria. Although, the gamma index results show distinct contrast of dose distribution outside the planning target volume (PTV) in IMRT, but within the PTV, it was acceptable. All three images could be used for OAR delineation and dose calculation in lung cancer. AIP image sets seemed to be suitable for dose calculation while patient movement between series acquisition of FB images should be considered when defining target volumes on 4DCT images.

## 1 Introduction

Advances in radiation therapy, radiation doses can now be tightly conformed to target volumes while minimizing dose delivery to surrounding normal organs.^1^, ^2^ Respiratory motion is a significant and challenging problem in radiation therapy due to geometrical uncertainties of both the target and normal organs in the thoracoabdominal region.^3^ The motion can potentially cause underdosage to tumor and overdosage to normal organs, and significantly diverted the planned and the delivered doses.^4^ Several approaches have been developed to manage the effects of respiratory motion during radiation therapy.^5^, ^6^ One of them is a motion‐encompassing method, which addresses the entire range of tumor motion and adds a margin to the target volumes. For dose calculation in this method, Vinogradskiy et al.^7^ and Guckenberger et al.^8^ compared three‐dimensional (3D) and four‐dimensional (4D) dose calculations and revealed minimal dosimetric differences in the gross tumor volume and the internal target volume (ITV). Thus, 3D dose calculation is still required in radiation therapy. For 3D radiation treatment planning in lung cancer, the ITV was delineated on maximum intensity projection (MIP) images, whereas organ at risk (OAR) contouring and static 3D dose calculation were done with 3DCT images.^9^, ^10^, ^11^, ^12^

3DCT images were also used to define OARs and targets for radiation therapy treatment, but the use of static images of a moving organ remains problematic.^13^ A common approach is to acquire helical computed tomography (CT) scans during free breathing (FB). Respiration‐induced target motion during acquisition, however, can cause motion artifacts.^14^, ^15^, ^16^ Several groups have demonstrated that dose calculation on average intensity projection (AIP) images, with each pixel holding the average value of all equivalent pixels in the data set, can yield results comparable to those attained with 4D dose calculation.^17^, ^18^, ^19^, ^20^ Others have proposed that mid‐ventilation (MidV) CT images represent the tumor in its time‐averaged position over the respiratory cycle and can be used for dose calculation. This position is an appropriate representation of the mean geometry and density of the target volumes for respiration‐induced anatomical variations.^21^, ^22^, ^23^, ^24^

This study was designed to compare three types of CT imaging (AIP, FB, MidV) for contouring and radiation treatment planning for lung cancer using 3D conformal radiation therapy (3DCRT) and intensity‐modulated radiation therapy (IMRT). Their differences are compared in terms of the OAR volume, geometrical center of the OARs, dosimetric indices, and dose distribution with regard to tumor size.

## 2 Materials and methods

### 2.A Patient selection

A total of 31 patients with various stages of lung cancer were enrolled in this study between January 2012 and December 2013. Our institutional review board approved the study protocol. Two patients were excluded because of their movement between the FB and 4DCT acquisitions.

### 2.B CT scanning for treatment planning

The patients were positioned in an immobilization device with a wing board on the scanner table, in the supine position with arms raised above the head (the treatment position). CT images of the thorax were acquired as normal FB and 4DCT scans using a helical 16‐slice Brilliance Big Bore CT scanner (Philips Medical Systems, Cleveland, OH, USA). The parameters for image acquisition were 120 kVp, 400 mAs, 512 × 512 matrix, 3.0‐mm slice thickness, and 0.5 s per rotation, with pitch values according to the manufacturer's recommendation for the particular respiratory rate of each patient. During CT image acquisition, the respiratory signal was recorded using either a Philips bellows system or a real‐time position management respiratory gating system (Varian Medical System, Palo Alto, CA, USA) synchronized with the CT data. For the 4DCT images, respiratory signal data were reconstructed and sorted into 10 equidistant time‐percentage bins (0% at maximum inhalation to 90%) throughout a respiratory cycle, each reflecting 10% of the respiratory cycle. Thus, the 0% respiratory phase corresponded to peak inhalation and the 50% respiratory phase corresponded to peak exhalation. MIP and AIP images were reconstructed from 10‐phase 4DCT data relating to the percentage of time. MidV images (representing the tumor in its time‐averaged position over the respiratory cycle) were chosen from the 10 phases of the 4DCT image data set. To make them appropriate for clinical use, the 4DCT images during the exhalation phase were displayed. We then selected the data set image that was closest to the central position of the tumor. All CT images (AIP, FB, MidV, MIP, 10‐phase 4DCT) were then transferred to a radiation treatment planning system (Eclipse, version 10.0.42; Varian Medical System) with a DICOM protocol connection.

### 2.C Delineation of the target and OARs

An experienced radiation oncologist generally delineated the planning target volume (PTV) and OARs are created by a geometric expansion based on the setup methods and the institutional guidelines. For each patient, the ITV was defined on the MIP images. The 10‐phase 4DCT was used to verify that the target motion was contained within the ITV during all phases. Each patient's PTV was obtained by adding 5 mm of circumferential expansion from the ITV and then copied to the AIP, FB, and MidV images. For these images, the OARs were delineated according to the guidelines provided by our respective protocols. The OARs used for plan comparisons in this study included the lung, trachea, heart, esophagus, and spinal cord, which are also shown on the images. The lung and trachea (including the main bronchus) were delineated with lung windows. Both lungs were automatically segmented using a threshold algorithm in the treatment planning system. The rest were contoured using mediastinal windows. The spinal cord was defined based on the bony limits of the spinal canal. The heart was delineated along with the pericardial sac starting below the level at which the pulmonary trunk branched into the left and right pulmonary arteries and extending inferiorly to the heart apex. The esophagus was defined from the bottom of the cricoid to the gastroesophageal junction.^14^

### 2.D OAR geometrical center

To identify an OAR's geometrical center, the OAR's volume was first determined from a CT image. The geometrical center of the OAR was then calculated in the Eclipse treatment planning system. The centroid of the volume coordinates for each OAR was measured from the radiation beam isosenter. The coordination was displayed as x, y, and z, which refer, respectively, to displacement in the mediolateral (ML), anteroposterior (AP), and superoinferior (SI) directions. The 3D vector was calculated for comparing the centroid of the OAR's volume in each image. The difference of the geometrical center of FB and MIP image sets comparison with respect to AIP image sets was calculated and analyzed.

### 2.E Radiation treatment planning

Based on AIP images obtained during the planning stage, the isocenter was within the tumor at the approximately geometrical center of the PTV. The treatment plan was calculated on a 2.0‐mm grid using an analytical anisotropic algorithm for dose calculation. The algorithm took into account the tissue inhomogeneity of the patient's body volume. The forward conventional 3D conformal and inverse IMRT techniques were applied. For 3DCRT planning, four to seven coplanar static 10‐MV photon beams were created for each patient using the beam's eye view technique. The beam arrangement was customized for each patient based on tumor location and nearby OARs. The multileaf collimators (MLCs) were conformed to the PTV with a 0.5‐cm margin around the PTV, except in the SI direction, where a 10‐mm margin was set. Beam weights and wedge angles were adjusted to provide homogeneous dose distribution and conform to the PTV. Total prescription doses were 50–66 Gy, with 2 Gy per fraction for the dose to be delivered to 95% of the PTV. Plans were manually optimized to meet all tumors and OAR objectives. The dose constraints to the OARs included the mean lung dose (lung minus ITV), which was < 20 Gy. The maximum dose to the spinal cord was < 50 Gy, the mean heart dose was < 35 Gy, and the maximum dose to the esophagus was < 105% of the prescribed dose.

The plans based on AIP images were subsequently used for comparisons with their respective MidV and FB image plans. The beam angle, beam modifier, field size, collimation, and beam weighting used in the AIP image plan were copied to the FB and MidV images, where they were re‐calculated. For IMRT planning recalculation, the plans were re‐optimized with the same objective and priority from the AIP plans. Re‐optimization and dose‐volume calculation in IMRT were performed with a sliding window MLC mode using a dose‐volume optimization algorithm and an analytical anisotropic algorithm, respectively. Both of these plans were normalized with the same 95% PTV dose coverage as was used for the AIP image plan.

In addition to the PTV, ring structures were created around the target structures to help force the optimization to minimize doses to surrounding OARs. Three control structures were created. The first and second control structures were 1‐ and 2‐cm rings around the PTV. The dose constraints of these structures were defined as < 90% and < 80% of the PTV prescribed dose, respectively. The third control structure was an outer ring that encompassed all tissues beyond the second ring. Only 50% of the PTV dose was assigned to this area. For an OAR that overlapped with the PTV, a virtual structure was created in which 5 mm of the OAR was subtracted from the PTV. Optimization dose constraints and priorities based on tolerance to the dose were initially applied. They were adjusted as the optimization process progressed to improve target coverage and reduce the OAR dose. When satisfied with the plan derived from the AIP image, it was copied and recalculated for the FB and MidV images with the same beam direction, dose constraints, and priorities. These plans were normalized with the same 95% PTV dose coverage as was used for the AIP imaging plan.

### 2.F Plan comparison and statistical analyses

The volume of each OAR and the centroid of each OAR's volume were measured and recorded. The impact of target volume size was studied with separation of small and large volume using volume cut‐off at 150 cm^3^. For dosimetric indices comparisons, the maximum (Dmax) and mean (Dmean) doses for each OAR were determined. MapCHECK software (Sun Nuclear Corporation, Melbourne, FL, USA) was used to evaluate the dose distribution. Planar dose analyses were then performed in the axial, sagittal, and coronal planes passing through the isosenter. Each plane of the dose distribution was transferred from the Eclipse treatment planning system (30 × 30 cm field of view, 512 × 512 matrix) to the MapCHECK software. The dose differences in each data set of the three image planes were then compared (AIP vs MidV, AIP vs FB, FB vs MidV). The dose distribution was analyzed to calculate the percentage of pixels passing gamma using a gamma index analysis with 2% dose difference and a 2‐mm distance‐to‐agreement criterion (2%/2 mm). Although, the report of the AAPM Task Group 119^25^ reported that the 3%∕3 mm gamma criteria is the most commonly used by clinical IMRT but higher sensitivity was observed with 2%/2 mm than with 3%/3 mm.^26^ All the data points with doses < 10% and 70% of the normalized dose were excluded from the gamma analysis for considering the difference of the low‐ and high‐dose regions, respectively. A gamma passing rate of 100% was considered strong agreement of the plans, and 0% was regarded as total disagreement.

Data analysis to compare the OARs’ volumes, the centroid of the OARs’ volumes, and dosimetric indices of the various CT images was performed using a Wilcoxon Signed Ranks test from the Statistical Package of Social Sciences (SPSS version 18.0; SPSS Inc., Chicago, IL, USA). Statistical significance was indicated at *P* < 0.05.

## 3 Results

In all, 31 lung cancer patients underwent FB and 4DCT simulations. The patients’ PTV information regarding target location, PTV volume, and target motion amplitude in the SI, ML, and AP directions as well as the prescribed dose and number of fractions were recorded. They are shown in Table 1.

**Table 1:** Patients’ PTV information and planned dose prescription

Patient no.	Target location	PTV volume (cm^3^)	Target motion amplitude (cm)			Prescribed dose (Gy)	Number of fractions
			SI	ML	AP		
1	RLL	368.7	1.00	0.54	0.55	60	30
2	LLL	358.4	0.30	0.15	0.34	60	30
3	LLL	400.8	0.27	0.38	0.10	54	27
4	RUL	347.7	0.46	0.47	0.52	66	33
5	RUL	315.1	0.30	0.20	0.20	60	30
6	RLL	43.9	1.25	1.14	0.37	66	33
7	RUL	35.5	0.95	0.26	0.17	66	33
8	LUL	948.1	0.20	0.10	0.10	66	33
9	RUL	449.9	0.42	0.00	0.15	66	33
10	RUL	315.6	0.48	0.14	0.25	66	33
11	RUL	280.2	0.40	0.10	0.20	60	30
12	LLL	187.3	0.20	0.10	0.10	66	33
13	RLL	110.0	0.58	0.32	0.10	66	33
14	RLL	71.3	0.37	0.24	0.10	66	33
15	LLL	184.0	0.92	0.33	0.15	66	33
16	LUL	286.0	0.26	0.15	0.10	50	25
17	LUL	96.5	0.40	0.20	0.20	60	30
18	RLL	298.1	0.80	0.20	0.10	60	30
19	RLL	221.0	0.90	0.60	0.20	60	30
20	LLL	10.0	0.49	0.30	0.23	60	30
21	RUL	606.7	0.57	0.33	0.24	50	25
22	RLL	298.3	0.40	0.10	0.10	50	25
23	LUL	127.9	0.40	0.16	0.10	60	30
24	RUL	33.4	0.60	0.20	0.24	60	30
25	LUL	290.2	0.10	0.10	0.10	60	30
26	RLL	221.1	0.40	0.10	0.30	60	30
27	RUL	48.1	0.90	0.40	0.14	60	30
28	RUL	22.6	0.60	0.50	0.20	66	33
29	LUL	425.1	0.40	0.20	0.20	50	25
30	LUL	705.0	0.55	0.30	0.22	60	30
31	RLL	349.1	0.82	0.60	0.35	50	25
Average (range)		272.77 (10.30–948.10)	0.54 (0.1–1.25)	0.29 (0–1.14)	0.21 (0.1–0.55)		

PTV, planning target volume; SI, superoinferior; ML, mediolateral; AP, anteroposterior; RLL, right lower lobe; LLL, left lower lobe; RUL, right upper lobe; LUL, left upper lobe.

According to the findings, tumors were found in the upper lobes in 17 patients and in the lower lobes in 14 patients. PTV volumes were in the range of 10.30–948.10 cm^3^. Tumor motion amplitudes were 0.10–1.25 cm in the SI direction, 0–1.14 cm in the ML direction, and 0.10–0.55 cm in the AP direction. Two patients (Patient no. 4 and 5) were later excluded from the study because they moved during the CT simulation process. The movement resulted in unmatched FB and 4DCT images in the series (based on bony anatomy), which could be a potential source of error. For instance, with the 3DCRT technique (not shown in the results), patient 4 had a huge deviation in the maximum dose to the esophagus. The dose was recorded as 63.11 Gy in the FB image plan; whereas it was 29.32 in the AIP plan and 29.17 in the MidV plan. For patient 5, the maximum dose to the spinal cord was 58.03 Gy in the FB image plan; whereas it was 49.16 in the AIP plan and 48.37 in the MidV plan.

**Figure 1:**
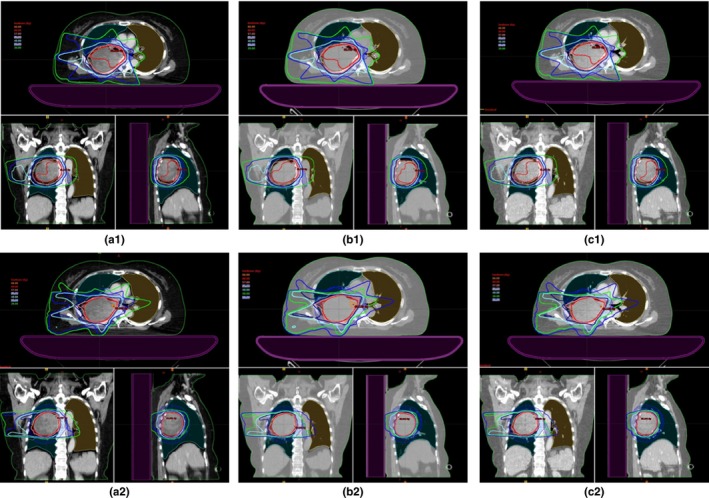
Isodose distributions in a patient as shown by three types of CT imaging of AIP images plan (a1 & a2), FB image plan (b1 & b2), and MidV image plan (c1 & c2) in 3DCRT (top row) and IMRT (bottom row). Orange = 66 Gy, red = 60 Gy, pink = 57 Gy, dark blue = 50 Gy, cyan = 40 Gy, blue = 30 Gy, green = 20 Gy.

To evaluate the dosimetric differences between the calculated plans from the three types of CT images, the AIP image plan was copied to the FB and MidV images and recalculated using the same beam arrangement and PTV coverage. Figure 1 represents the comparison between the three plans to illustrate the dose distributions for 3DCRT and IMRT.

The volumes for OAR contouring in the remaining 29 patients are shown in Table 2. There were no significant differences in the OARs’ dose volumes for both small and large target volumes. The average volumes from the AIP, FB, and MidV images were 2832.66, 2906.34, and 2810.81 cm^3^, respectively, for small targets and 2342.69, 2317.95, and 2318.57 cm^3^ for large targets, respectively.

**Table 2:** OARs volumes by three types of CT imaging

OARs	Tumor size	CT images	Volume (cm^3^)	Comparison	*P*
Lungs‐ITV	Small PTV	AIP	2832.66 ± 1043.00	AIP vs FB	0.241
		FB	2906.34 ± 1009.79	AIP vs MidV	0.075
		MidV	2810.81 ± 1030.88		
	Large PTV	AIP	2342.69 ± 797.50	AIP vs FB	0.184
		FB	2317.95 ± 782.67	AIP vs MidV	0.879
		MidV	2318.57 ± 790.72		
Trachea	Small PTV	AIP	41.39 ± 11.95	AIP vs FB	0.575
		FB	41.63 ± 12.22	AIP vs MidV	0.386
		MidV	40.88 ± 11.84		
	Large PTV	AIP	41.21 ± 14.35	AIP vs FB	0.619
		FB	40.43 ± 14.05	AIP vs MidV	0.984
		MidV	41.56 ± 12.85		
Heart	Small PTV	AIP	719.22 ± 237.89	AIP vs FB	0.799
		FB	726.11 ± 223.14	AIP vs MidV	0.959
		MidV	720.24 ± 235.15		
	Large PTV	AIP	627.87 ± 104.05	AIP vs FB	0.084
		FB	619.87 ± 108.70	AIP vs MidV	0.171
		MidV	623.25 ± 110.41		
Esophagus	Small PTV	AIP	44.55 ± 12,58	AIP vs FB	0.959
		FB	44.58 ± 12.76	AIP vs MidV	0.799
		MidV	44.56 ± 12.56		
	Large PTV	AIP	39.23 ± 8.19	AIP vs FB	0.064
		FB	39.58 ± 8.16	AIP vs MidV	0.117
		MidV	39.59 ± 8.16		
Spinal cord	Small PTV	AIP	39.08 ± 16.19	AIP vs FB	0.208
		FB	39.29 ± 16.06	AIP vs MidV	0.139
		MidV	39.34 ± 16.22		
	Large PTV	AIP	35.96 ± 8.82	AIP vs FB	0.494
		FB	36.19 ± 8.70	AIP vs MidV	0.324
		MidV	36.15 ± 8.88		

Results are reported as the average ± SD.

The difference of magnitudes in vector geometrical center (3D vectors) between the reference AIP image sets with respect to the test image sets (FB and MidV) are shown in Table 3. There were no significant differences in both tumor sizes in the centers of the OARs’ volumes (*P* > 0.05) between AIP image and test image sets.

**Table 3:** OARs geometric center difference between AIP and FB and MidV CT imaging

OARs	Conditions		3D vector difference (mm)			*P*
			Average	Min.	Max.	
Lungs‐ITV	Small PTV	AIP vs FB	−0.04	−0.61	0.18	0.799
		AIP vs MIP	0.03	−0.07	0.18	0.445
	Large PTV	AIP vs FB	−0.05	−0.78	0.52	0.658
		AIP vs MIP	0.06	−0.34	0.57	0.084
Trachea	Small PTV	AIP vs FB	0.02	−0.15	0.22	0.721
		AIP vs MIP	−0.01	−0.13	0.20	0.799
	Large PTV	AIP vs FB	0.02	−0.22	0.28	0.601
		AIP vs MIP	−0.03	−0.22	0.17	0.133
Heart	Small PTV	AIP vs FB	0.02	−0.19	0.25	0.799
		AIP vs MIP	0.01	−0.17	0.18	0.799
	Large PTV	AIP vs FB	−0.06	−0.27	0.23	0.078
		AIP vs MIP	−0.02	−0.40	0.31	0.546
Esophagus	Small PTV	AIP vs FB	0.00	−0.71	0.34	0.441
		AIP vs MIP	0.01	−0.28	0.21	0.735
	Large PTV	AIP vs FB	−0.09	−0.54	0.39	0.171
		AIP vs MIP	−0.02	−0.44	0.37	0.520
Spinal cord	Small PTV	AIP vs FB	−0.01	−0.21	0.13	0.799
		AIP vs MIP	0.02	−0.05	0.10	0.575
	Large PTV	AIP vs FB	−0.06	−0.49	0.17	0.227
		AIP vs MIP	−0.01	−0.28	0.24	0.809

OARs, organs at risk; AIP, average intensity projection; FB, free breathing; MidV, mid‐ventilation.

The averages and standard deviations of the maximum doses for each OAR determined from the CT images are summarized in Table 4. For 3DCRT, there were no significant differences in the maximum dose of any of the OARs (lung – ITV, trachea, heart, esophagus, spinal cord) among the three plans. For IMRT, there were also no significant differences in the maximum doses for the OARs among the three plans.

**Table 4:** Maximum doses to the OARs seen by three types of CT imaging

OARs	Tumor size	CT images	Maximum dose (Gy)		Comparison	*P*	
						3DCRT	IMRT
			3DCRT	IMRT			
Lungs‐ITV	Small PTV	AIP	66.01 ± 3.73	66.39 ± 3.73	AIP vs FB	0.879	0.721
		FB	61.66 ± 4.12	66.59 ± 3.53	AIP vs MidV	0.285	0.075
		MidV	66.13 ± 3.73	66.56 ± 3.64			
	Large PTV	AIP	60.70 ± 6.03	62.81 ± 7.18	AIP vs FB	0.212	0.260
		FB	60.68 ± 6.11	63.02 ± 7.14	AIP vs MidV	0.794	1.00
		MidV	60.68 ± 6.04	62.82 ± 7.21			
Trachea	Small PTV	AIP	30.64 ± 26.95	25.79 ± 1.71	AIP vs FB	0.386	0.647
		FB	31.37 ± 26.93	25.83 ± 25.72	AIP vs MidV	0.203	0.386
		MidV	30.78 ± 26.92	24.12 ± 25.47			
	Large PTV	AIP	46.53 ± 22.08	45.32 ± 23.62	AIP vs FB	0.137	0.990
		FB	46.72 ± 22.04	45.93 ± 23.58	AIP vs MidV	0.778	0.091
		MidV	46.57 ± 22.11	45.87 ± 23.58			
Heart	Small PTV	AIP	42.83 ± 24.88	28.63 ± 27.93	AIP vs FB	0.241	0.575
		FB	42.56 ± 24.99	27.93 ± 27.82	AIP vs MidV	0.285	0.075
		MidV	42.65 ± 24.77	28.12 ± 27.66			
	Large PTV	AIP	45.15 ± 22.49	45.24 ± 24.72	AIP vs FB	0.593	0.809
		FB	43.28 ± 22.56	45.22 ± 24.63	AIP vs MidV	0.070	0.872
		MidV	45.33 ± 22.30	45.37 ± 25.06			
Esophagus	Small PTV	AIP	36.41 ± 23.51	34.26 ± 23.16	AIP vs FB	0.285	0.241
		FB	34.81 ± 21.61	33.97 ± 22.95	AIP vs MidV	0.879	0.285
		MidV	36.82 ± 22.20	34.46 ± 22.95			
	Large PTV	AIP	48.69 ± 16.29	51.68 ± 15.21	AIP vs FB	0.260	0.376
		FB	48.76 ± 16.55	51.39 ± 15.44	AIP vs MidV	0.243	0.872
		MidV	48.54 ± 16.26	51.76 ± 15.17			
Spinal cord	Small PTV	AIP	25.51 ± 14.42	19.06 ± 9.99	AIP vs FB	0.333	0.114
		FB	25.99 ± 14.62	19.24 ± 9.90	AIP vs MidV	0.445	0.241
		MidV	25.85 ± 14.64	19.02 ± 9.92			
	Large PTV	AIP	41.62 ± 10.70	36.00 ± 6.07	AIP vs FB	0.469	0.421
		FB	41.70 ± 10.43	36.17 ± 5.84	AIP vs MidV	0.520	0.841
		MidV	41.36 ± 10.45	36.03 ± 5.32			

Results are reported as the average ± SD.

The averaged values and standard deviations of the mean doses for the OARs are shown in Table 5. No significant differences (*P* > 0.05) were found for the OARs in all image data sets with both 3DCRT and IMRT.

**Table 5:** Mean dose to the OARs on three types of CT imaging

OARs	Tumor size	CT images	Mean dose (Gy)		Comparison	*P*	
						3DCRT	IMRT
			3DCRT	IMRT			
Lungs‐ITV	Small PTV	AIP	7.57 ± 2.57	5.85 ± 1.75	AIP vs FB	0.374	0.169
		FB	7.26 ± 2.47	5.58 ± 1.77	AIP vs MidV	0.721	0.114
		MidV	7.59 ± 2.51	5.79 ± 1.71			
	Large PTV	AIP	15.53 ± 4.39	12.67 ± 4.82	AIP vs FB	0.940	0.546
		FB	15.56 ± 4.67	12.79 ± 5.24	AIP vs MidV	0.117	0.147
		MidV	15.73 ± 4.53	12.89 ± 4.89			
Trachea	Small PTV	AIP	8.23 ± 9.24	6.14 ± 6.89	AIP vs FB	0.800	0.779
		FB	8.11 ± 8.84	6.04 ± 6.59	AIP vs MidV	0.169	0.169
		MidV	7.93 ± 8.67	5.95 ± 6.61			
	Large PTV	AIP	23.61 ± 15.54	19.82 ± 13.72	AIP vs FB	0.904	0.809
		FB	23.83 ± 15.94	19.92 ± 13.90	AIP vs MidV	0.766	0.171
		MidV	23.12 ± 15.46	19.49 ± 13.84			
Heart	Small PTV	AIP	5.52 ± 4.98	2.33 ± 2.72	AIP vs FB	0.386	0.285
		FB	5.67 ± 5.21	2.31 ± 2.70	AIP vs MidV	0.241	0.333
		MidV	5.62 ± 5.13	2.28 ± 2.67			
	Large PTV	AIP	11.44 ± 7.10	7.25 ± 4.82	AIP vs FB	0.711	0.904
		FB	11.62 ± 7.12	7.25 ± 4.83	AIP vs MidV	0.468	0.658
		MidV	11.66 ± 7.03	7.37 ± 4.75			
Esophagus	Small PTV	AIP	8.51 ± 4.84	6.67 ± 4.61	AIP vs FB	0.647	0.721
		FB	8.46 ± 4.76	6.77 ± 4.90	AIP vs MidV	0.799	0.139
		MidV	8.51 ± 4.78	6.83 ± 4.66			
	Large PTV	AIP	18.98 ± 7.85	16.94 ± 7.15	AIP vs FB	0.809	0.398
		FB	19.33 ± 8.87	16.60 ± 7.92	AIP vs MidV	0.260	0.277
		MidV	19.46 ± 8.11	16.74 ± 7.21			
Spinal cord	Small PTV	AIP	9.72 ± 17.72	2.77 ± 1.25	AIP vs FB	0.386	0.647
		FB	9.54 ± 16.30	2.79 ± 1.26	AIP vs MidV	0.285	0.959
		MidV	9.62 ± 16.96	2.79 ± 1.31			
	Large PTV	AIP	11.77 ± 4.24	8.90 ± 2.71	AIP vs FB	0.398	0.617
		FB	11.86 ± 4.44	8.97 ± 2.79	AIP vs MidV	0.184	0.629
		MidV	11.53 ± 4.44	8.98 ± 2.72			

Results are reported as the average ± SD.

The results of the gamma index analyses in 29 patients are shown in Table 6. Two‐dimensional (2D) dose distributions were calculated on three orthogonal planes at the isosenter. An average gamma passing rate > 98% (2%, 2 mm) was obtained for all investigated scenarios using 3DCRT. For IMRT, the 2D dose distribution agreement had a higher than 90% gamma passing rate. As a result of gamma passing rate with 10% and 70% cut‐off levels, high gamma passing rate at 70% has a value more than 10% cut‐off level. This indicated that the high‐dose region appeared to be in agreement with dose distribution for both 3DCRT and IMRT.

**Table 6:** Gamma passing rate derived from a comparison of the 2D dose distribution according to three types of CT imaging on three orthogonal planes at the isocenter

CT type and image plane	% Cut‐off	Tumor size	Gamma passing rate (%)	
			3DCRT	IMRT
AIP vs FB				
Axial	10%	Small PTV	98.9 ± 1.6	95.8 ± 5.3
		Large PTV	99.5 ± 0.7	94.3 ± 9.4
	70%	Small PTV	99.3 ± 1.9	97.4 ± 5.1
		Large PTV	99.9 ± 0.3	98.4 ± 3.7
Coronal	10%	Small PTV	99.3 ± 0.2	98.6 ± 2.5
		Large PTV	99.2 ± 1.9	95.9 ± 6.2
	70%	Small PTV	99.5 ± 1.2	98.6 ± 2.7
		Large PTV	99.7 ± 1.1	97.9 ± 3.3
Sagittal	10%	Small PTV	98.4 ± 2.8	96.6 ± 3.8
		Large PTV	99.4 ± 0.9	96.1 ± 7.3
	70%	Small PTV	98.6 ± 2.8	98.1 ± 4.6
		Large PTV	99.6 ± 1.1	97.9 ± 3.0
AIP vs MidV				
Axial	10%	Small PTV	100 ± 0.0	96.6 ± 5.8
		Large PTV	99.9 ± 0.3	91.9 ± 14.1
	70%	Small PTV	100 ± 0.0	99.6 ± 0.7
		Large PTV	100 ± 0.0	96.5 ± 5.9
Coronal	10%	Small PTV	100 ± 0.0	98.7 ± 1.9
		Large PTV	99.9 ± 0.3	92.6 ± 13.8
	70%	Small PTV	100 ± 0.0	100 ± 0.1
		Large PTV	100 ± 0.0	98.2 ± 3.6
Sagittal	10%	Small PTV	100 ± 0.1	99.1 ± 1.3
		Large PTV	100 ± 0.0	95.6 ± 9.9
	70%	Small PTV	100 ± 0.0	99.9 ± 0.2
		Large PTV	100 ± 0.0	98.9 ± 2.4

Results are reported as the average ± SD.

## 4 Discussion

Several studies have focused on 3DCT images for radiation treatment planning on moving targets. In most cases, stereotactic body radiation therapy (SBRT) planning was investigated. Our study compared conventional radiation doses using 3DCRT and IMRT to determine the usefulness of three types of CT imaging for radiation treatment planning. For 3DCRT, there is no change in the radiation beam parameters (beam fluence), unlike when using advanced techniques such as IMRT or volumetric modulated arc therapy. For SBRT, however, the PTV volume is smaller than that needed for 3DCRT. In addition, the differences in OARs’ dose volumes and dosimetric indices are evaluated using the same plan as for PTV dose coverage.

Our study was similar to that of Han et al.,^14^ who compared AIP and FB images regarding OAR contouring and radiation treatment (SBRT) planning for 10 lung cancer patients. They found differences in the volumes of lungs – ITV but no significant differences in the rest of the OAR volumes. Tian et al.^13^ compared the dosimetric indices during treatment (SBRT) planning calculated using FB, MIP, and AIP images for 20 lung cancer patients. They observed no significant differences in dosimetric indices for PTVs and the lung between the FB and AIP plans. We found limitations in the use of FB images for planning radiation treatment. We demonstrated patient movement between series acquisition using 4DCT and FB imaging to delineate the target dose volume. Two patients were excluded as they moved during the CT simulation process, invalidating the data. When comparing the planning of AIP vs FB and FB vs MidV, the 2%/2 mm average gamma passing rates of the 2D dose distribution were less than 90% on three orthogonal planes at the isocenter. For 3DCRT, the 2D dose distribution using three types of CT imaging in three orthogonal planes at the isocenter location in 29 patients showed good dose distribution agreement between AIP and MidV plans, with a higher than 98% gamma passing rate. The gamma passing rates were lower when dose distributions of AIP vs FB planning were compared. The difference may be due to the use of different types of CT imaging, which could produce density variations induced by movement effects. Furthermore, the FB images were of lower quality than the AIP and MidV images because of motion artifacts as reported in study of Han et al. and Tian et al. The motion artifacts can cause axial slices being shuffled out of order and organs being imaged as distinct parts, distorted, or displaced, resulting in errors associated with the target volume, OAR delineation, and dose calculation. FB acquisition was also undertaken with an increased imaging dose. FB images are still used by some groups, because they can more clearly distinguish the target volume from contrast agent filtration into the tumor than in 4DCT images. As acquisition of 4DCT images may require a long period, the contrast agent inside the tumor becomes less evident. The two imaging data sets from 4DCT acquisitions (AIP and MidV) were obtained for OAR delineation and dose calculation using only one CT acquisition. This capability can reduce one source of error from the radiation treatment planning process. Overall, for each percentage of the gamma passing rate, the IMRT dose was lower than that with 3DCRT. The results also showed that most of the dose difference was outside the PTV volume. This result can be explained by considering the basic concept of the IMRT technique—that the intensity of the dose was modulated throughout the radiation field. Thus, a comparison of the doses in each plane showed that the difference diminished as one approached the target volume. For impact of target size to dosimetric parameters, this study found that no difference for either small and large targets in terms of OAR volumes, geometrical center difference of OARs, maximum and minimum dose of OARs and dose distribution.

There was also good agreement between dose distributions in PTV from different CT images as shown by the gamma index analysis. There were also no significant differences in the geometrical center of the OARs. Hence, it is likely that all three images could be used for radiation treatment planning for a moving target. Nonetheless, determining the effects of patient movement on FB images is crucial.

Further study is needed to evaluate the accuracy of dose delivery to patients. As radiation treatment planning represents the actual dose distribution, it is necessary to verify dose distribution in a moving target volume compared with dose distribution in treatment planning.

## 5 Conclusion

Radiation treatment planning according to the data sets derived from the three types of CT imaging (AIP, FB, MidV) using conventional 3DCRT and IMRT techniques for lung cancer patients revealed no significant difference in the OAR volumes and the geometric centers of the OARs. Our data also indicated good agreement of the 2D dose distributions for all the three plans, with a higher than 90% average gamma passing rate (2%, 2 mm) for 3DCRT and IMRT. Although the IMRT plan displayed dose differences outside the PTV, doses bordering to the PTV were in closer agreement with the targeted dose distribution. The dosimetric indices (maximum and mean doses) for all three plans, however, showed no significant differences among the OARs and the size of target volume. We also demonstrated that the AIP, FB, and MidV images could be used for OAR contouring and dose calculation. AIP image sets seemed to be suitable for contouring and dose calculation. It is essential to be aware of the variations in patient movement regarding the FB images because they affect delimitation of the target volume on 4DCT images.
